# Cardio-Metabolic Benefits of Plant-Based Diets

**DOI:** 10.3390/nu9080848

**Published:** 2017-08-09

**Authors:** Hana Kahleova, Susan Levin, Neal Barnard

**Affiliations:** 1 Physicians Committee for Responsible Medicine, 5100 Wisconsin Ave, N.W. Ste.400, Washington, DC 20016, USA; slevin@pcrm.org (S.L.); nbarnard@pcrm.org (N.B.); 2 George Washington University School of Medicine and Health Sciences, Washington, DC 20052, USA

**Keywords:** cardio-metabolic, diet, nutrition, plant-based, vegan, vegetarian

## Abstract

Cardio-metabolic disease, namely ischemic heart disease, stroke, obesity, and type 2 diabetes, represent substantial health and economic burdens. Almost one half of cardio-metabolic deaths in the U.S. might be prevented through proper nutrition. Plant-based (vegetarian and vegan) diets are an effective strategy for improving nutrient intake. At the same time, they are associated with decreased all-cause mortality and decreased risk of obesity, type 2 diabetes, and coronary heart disease. Evidence suggests that plant-based diets may reduce the risk of coronary heart disease events by an estimated 40% and the risk of cerebral vascular disease events by 29%. These diets also reduce the risk of developing metabolic syndrome and type 2 diabetes by about one half. Properly planned vegetarian diets are healthful, effective for weight and glycemic control, and provide metabolic and cardiovascular benefits, including reversing atherosclerosis and decreasing blood lipids and blood pressure. The use of plant-based diets as a means of prevention and treatment of cardio-metabolic disease should be promoted through dietary guidelines and recommendations.

## 1. Introduction

Cardio-metabolic disease, namely ischemic heart disease, stroke, obesity, and type 2 diabetes, represent substantial health and economic burdens [1]. Suboptimal nutrition is a leading contributor to chronic disease and premature death in the United States and worldwide [2,3]. According to a recent analysis, certain dietary factors, including high intakes of sodium and processed meat products and low intakes of fruits and vegetables, were associated with 45.5% of cardio-metabolic deaths in the United States [4].

In this review, we present evidence that plant-based diets (for the purposes of this paper, “plant-based” will serve as a substitution for vegetarian and vegan diets only) may be an effective strategy for improving nutrient intake [5]. Plant-based diets are associated with decreased all-cause mortality and decreased risk of obesity, type 2 diabetes, and coronary heart disease [6].

Plant-based diets are characterized by a reduction or elimination of animal product consumption. They are typically based on the consumption of grains, legumes, vegetables, fruits, and nuts. Vegan diets contain only plant foods, while lacto-ovo-vegetarian diets include dairy and/or egg products.

In this narrative review, we summarize the most recent findings on the effect of plant-based diets on cardio-metabolic disease risk. For each section, we searched for papers with the following key words: plant-based, vegetarian and vegan, giving special attention to systematic reviews and meta-analyses, particularly those drawing on randomized clinical trials. Findings from observational studies were included as supporting evidence.

## 2. Plant-Based Diets and Cardiovascular Disease

Cardiovascular disease is the leading cause of mortality, accounting for one in four deaths worldwide [7]. The high prevalence of heart disease has been linked to lifestyle factors, namely smoking, the adoption of diets high in animal fat and refined foods, and a lack of exercise [8].

A low-fat, vegetarian diet is the only dietary pattern to have shown cessation and reversal of atherosclerotic plaque in clinical trials [9,10,11], when combined with exercise and stress management [10,12,13]. Vegetarian diets are associated with a reduced risk for cardiovascular disease in general [14,15], including a reduced risk for ischemic heart disease and cerebrovascular disease [16]. Risk factors associated with heart disease are also less frequent among those following vegetarian diets [17,18]. In the European Prospective Investigation into Cancer and Nutrition study, vegetarians had a 32% lower risk of developing coronary heart disease, compared with non-vegetarians [15].

In a systematic review and meta-analysis of 8 prospective studies among Seventh-day Adventists, vegetarian diets were associated with a 40% reduced risk of coronary heart disease events and a 29% reduction in cerebral vascular disease events, compared with non-vegetarians [16]. A recent systematic review and meta-analysis of 86 cross-sectional and 10 cohort prospective studies reported a significant protective effect of a vegetarian diet against the incidence and/or mortality from ischemic heart disease. The observed risk reduction, compared with non-vegetarian dietary patterns, was 25% [19].

In summary, strong and consistent evidence from randomized clinical trials and observational studies supports beneficial effects of plant-based diets for cardiovascular disease.

## 3. Plant-Based Diets, Body Weight, and Metabolic Syndrome

The prevalence of overweight and obesity is increasing worldwide. The World Health Organization estimates that more than 1.3 billion adults worldwide are overweight, and a further 600 million are obese [20,21]. Overweight and obesity are associated with higher all-cause mortality [22].

Vegetarians typically have lower BMI (body mass index) values, compared with non-vegetarians [23]. BMI values tend to increase with increasing frequency of animal product consumption. In the Adventist Health Study-2, BMIs were lowest among vegans (23.6 kg/m^2^), higher in lacto-ovo-vegetarians (25.7 kg/m^2^), and highest in nonvegetarians (28.8 kg/m^2^) [24,25,26]. The average individual yearly weight gain is reduced when people limit consumption of animal foods [27]. Vegetarian diets seem to increase resting energy expenditure [28], which may be partly responsible for the lower BMI values in vegetarians.

Plant-based diets have been shown to be a particularly effective dietary approach for weight loss [29,30]. A recent study showed a mean BMI reduction of 4.4 kg/m^2^ with a 6-month, whole-food, plant-based diet with no energy restrictions, compared with usual care (0.4 kg/m^2^), in overweight or obese subjects [31].

In a meta-analysis of randomized trials by Huang et al., plant-based diets were associated with a mean weight reduction of −2.02 kg (95% confidence intervals (CI), −2.8 to −1.23 kg). A vegan diet had a more pronounced effect (−2.52 kg; 95% CI, −3.02 to −1.98 kg) than a lacto-ovo-vegetarian diet (−1.48 kg; 95% CI, −3.43 to 0.47 kg) [32]. Similarly, a meta-analysis of 15 clinical trials using vegetarian or vegan diets showed an average weight loss range of 4.6 kg among study completers [30].

Plant-based diets appear to reduce the risk of developing metabolic syndrome by about one half [33]. They reduce the risk of individual components of the metabolic syndrome (except for low high-density lipoprotein (HDL) cholesterol) and are associated with lower waist circumference [33], lower concentrations of triglycerides, total and low-density lipoprotein (LDL) cholesterol [33,34,35], blood sugar, and blood pressure [33,34].

Two recent meta-analyses of randomized clinical trials showed a benefit of plant-based diets for body weight. This is supported by the observational studies.

## 4. Plant-Based Diets and Glycemic Control

The prevalence of type 2 diabetes has been increasing worldwide. An estimated 382 million adults worldwide had diabetes in 2013; this number is expected to rise to 592 million by 2035 [36]. The economic burden associated with diabetes (both diagnosed and undiagnosed) exceeded $322 billion in 2012 in the United States [37]. Therefore, interventions to prevent and manage type 2 diabetes and its complications are desirable.

Diabetes prevalence has been found to be the lowest among vegans (Odds ratio (OR) 0.51; 95% CI 0.40–0.66) and lacto-ovo-vegetarians (OR 0.54; 95% CI 0.49–0.60), compared with non-vegetarians [26]. Diabetes incidence has also been observed to be the lowest in vegans (OR 0.381; 95% CI 0.236–0.617), lacto-ovo vegetarians (OR 0.618; 95% CI 0.503–0.760) and semi-vegetarians (OR 0.486, 95% CI 0.312–0.755). They all had a lower risk of diabetes than non-vegetarians [38].

Vegetarian diets have been shown to be helpful not only in prevention but also in the treatment of type 2 diabetes in several clinical trials. Early studies reported a dramatic reduction in the use of glucose-lowering medications and in plasma glucose levels, in response to a plant-based diet combined with exercise [39,40]. A 2014 meta-analysis found that a plant-based diet significantly improves blood sugar control in type 2 diabetes. The benefit of omitting meat, cheese, and eggs was as much as 0.7 points in some studies, and averaged about 0.4 points overall [41].

Even without exercise, beneficial effects of vegetarian diets included reduced body weight, better glycemic control, and lower blood lipids, compared with more conventional diets in treatment of type 2 diabetes—vegetarian diets being almost twice as effective [42,43,44].

In a long-term intervention study, the positive effects of a vegetarian diet (compared to a conventional reduced-energy diet) were partially preserved one year after the end of the intervention, although the patients did not continue with their originally assigned diets and consumed a comparable diet during that year [45]. A 2007 study showed that overweight participants following a low-fat, vegan diet were able to lose weight and keep most of it off after two years, more so than those following a diet based on the National Cholesterol Education Program guidelines [46].

Management of glycemic control is one of the cornerstones of diabetes care [47]. It has been well established that improved glycemic control reduces the risk of microvascular complications, whereas the role of glycemic control in reducing macrovascular complications is less clear.

Most observational studies have demonstrated a positive association between poor glucose control and the risk of cardiovascular disease [48,49,50,51,52,53,54]. Patients with HbA1c (glycated hemoglobin) concentrations of 6.0–6.9% had 20% lower relative risk of fatal/nonfatal coronary heart disease than patients with HbA1c concentrations of 7.0–7.9%. Limited data from four large, randomized, controlled trials and their follow-ups also suggest that chronic hyperglycemia is associated with an increased risk for cardiovascular disease in patients with diabetes [55,56,57,58,59,60,61,62,63,64]. Meta-analyses of these trials demonstrated significantly reduced risks of fatal/nonfatal myocardial infarction (15%) and cardiovascular disease (11–15%) with HbA1c reductions of approximately 1 absolute percentage point [58,65,66].

A recent meta-analysis of six randomized controlled trials showed that consumption of vegetarian diets was associated with a significant reduction in HbA1c by 0.4 absolute percentage points, compared with conventional diets in patients with type 2 diabetes [41]. This reduction in HbA1c alone (i.e., independently from improvements in body weight, blood lipids, blood pressure, platelet aggregation, and other variables) would be expected to decrease risks of myocardial infarction and cardiovascular disease by about 6% and 4.4–6%, respectively, based on estimates drawn from large prospective studies. Other healthful lifestyle factors add further reduction in risk.

One of the mechanisms that is likely responsible for improved glycemic control is increased insulin sensitivity in response to plant-based diets demonstrated in controlled trials [42]. It has also been demonstrated that partial replacement of meat with soy products increased insulin sensitivity in a randomized crossover trial [67].

Another potential mechanism responsible for improved glycemic control is improved gastrointestinal hormone response. Gastrointestinal hormones, especially the incretins, play an important role in postprandial increase in plasma insulin [68]. In patients with type 2 diabetes, the incretin effect is diminished [69], and it seems to be influenced by diet composition. Consumption of processed meat, for example, leads to impaired release of gastrointestinal hormones, including the incretins both in a fasting state and after a meal compared with an isocaloric vegan meal [70]. These results suggest that vegetarian diets may be beneficial for improvement in gastrointestinal hormone release in patients with type 2 diabetes.

In summary, the evidence for the beneficial effects of plant-based diets on glycemic control comes from six randomized controlled trials, summarized in a recent meta-analysis, as well as observational studies. Although the number of studies on this topic is limited, the concordance of results across studies is compelling.

## 5. Plant-Based Diets and Blood Pressure

It has been estimated that 874 million adults worldwide have a systolic blood pressure of 140 mm Hg or higher. In an analysis of data from 844 population-based studies in 154 countries between 1990 and 2015, 14% of all deaths and 143 million life-years of disability were attributable to hypertension [71].

In the United States, hypertension is associated with several leading causes of death, including heart disease, cancer, stroke, and diabetes [72]. Each 20 mm Hg increase in systolic blood pressure or each 10 mm Hg increase in diastolic blood pressure more than doubles the risk of death from stroke [73]. Conversely, a reduction of 5 mm Hg in systolic blood pressure leads to a 7 percent reduced risk of all-cause mortality, a 9 percent reduced risk of heart disease, and a 14 percent reduced risk of stroke [74]. High protein intake, especially from meat, increases blood pressure [75]. High potassium intake, however, lowers blood pressure among people with hypertension [76]. This may also be relevant in childhood in order to prevent hypertension in adulthood [77]. Vegetarian diets typically have higher fiber and potassium and lower fat, compared with omnivorous diets [5].

A meta-analysis of 7 randomized controlled trials and 32 observational studies found that vegetarian diets lower blood pressure (both systolic and diastolic), compared with omnivorous diets. In observational studies, vegetarian diets were associated with blood pressure readings that were, on average, 6.9 mm Hg and 4.7 mm Hg lower for systolic and diastolic blood pressure, respectively. In randomized controlled trials, vegetarian diets decreased both systolic and diastolic blood pressure by 4.8 and 2.2 mm Hg, respectively [78]. The blood pressure reduction was independent of salt intake, overweight, and exercise levels. The reduction in systolic blood pressure by 5 mm Hg is estimated to result in a 7% reduction in all-cause mortality, a 9% reduction in mortality due to coronary heart disease, and a 14% reduction in mortality due to stroke [74,78].

In summary, a recent meta-analysis of randomized clinical trials and observational studies showed clear benefits of plant-based diets for blood pressure. Given the consistent results between the studies, the evidence is strong.

## 6. Plant-Based Diets and Blood Lipids

Epidemiological studies have shown a high prevalence of hypercholesterolemia in Western countries (more than 50% adults have total cholesterol serum levels higher than 5 mmol/L), along with the high incidence of cardiovascular disease and related deaths [79,80,81]. Data from clinical studies indicate that for every 1% reduction in LDL-cholesterol, the risk for a major cardiac event, including heart attack and stroke, is reduced by approximately 1% [82]. Since lifestyle changes (especially diet and exercise) can lower LDL levels by 30–40% in people with or at risk for heart disease, reducing LDL-cholesterol to lower targets can play a significant role in disease prevention and possibly treatment [10,83].

Saturated fat increases plasma LDL cholesterol concentrations. According to a report published by the American Heart Association, replacing saturated fat in the diet and replacing it with polyunsaturated vegetable oil can reduce the risk of cardiovascular disease by about 30%, similar to the effect of statins. The authors concluded that the incidence of cardiovascular disease (CVD) would decrease with such a dietary shift [84].

The effect of dietary cholesterol on plasma cholesterol concentrations is less pronounced than that of saturated fat. Nonetheless, a recent meta-analysis confirmed the longstanding observation that dietary cholesterol increases serum total and LDL-cholesterol concentrations [85]. Dietary cholesterol is found only in animal products including meat, dairy, and eggs. A meat-free diet can lead to a significant reduction in total and LDL cholesterol, which corresponded with about a 10% reduced risk of heart disease, according to a meta-analysis of randomized-controlled trials published by the American Heart Association [17].

Vegetarian and especially vegan dietary patterns improve both fasting and postprandial blood lipids compared with conventional therapeutic diets [18,86,87,88,89,90,91], with effects similar to those seen with statin therapy [92]. If combined with moderate physical exercise, smoking cessation and stress management, the reduction of blood lipids can be even higher [10].

In summary, the findings of interventional trials are in accordance with those of observational studies, and the evidence for improved blood lipid profiles in response to plant-based diets is strong.

## 7. Plant-Based Diets and Platelet Aggregation

Enhanced platelet adhesion, activation, and aggregation increase the risk of ischemic stroke. In addition, insulin resistance plays a role in the pathogenesis of ischemic stroke by encouraging atherosclerotic changes. Clinical studies have suggested that improving insulin resistance may be an effective way how to prevent or delay ischemic stroke [88].

Both platelet aggregation and insulin resistance are influenced by diet choices. Plant-based diets have been shown to reduce insulin resistance [42], as well as to reduce platelet aggregation and thus reduce cardiovascular risk [89]. Plant foods with low glycemic index like whole grains, vegetables, nuts, legumes, garlic, ginger, onion, purple grape juice, tomatoes, berries, and dark chocolate, are particularly efficient in reducing platelet aggregation [89].

Because of the paucity of studies examining the effect of plant-based diets on platelet aggregation, the evidence for its beneficial effects is limited.

## 8. Potential Mechanisms Responsible for Benefits Associated with Plant-Based Diets

Several possible mechanisms may explain the beneficial cardio-metabolic effects of plant-based diets: lower caloric intake, increased intake of fiber, reduced intake of saturated fat and cholesterol, higher intake of polyunsaturated and monounsaturated fatty acids, increased intake of antioxidants and micronutrients, higher intake of vegetable protein, and a higher intake of plant sterols.

A reduction in energy intake due to the lower energy density of plant foods [93] has been shown to yield cardio-metabolic benefits even before any changes in body weight occur [93].

The ideal percentage carbohydrate, protein, and fat in the diet is a subject of ongoing discussion and debate. Plant-based diets used in the treatment of cardio-metabolic disease in clinical trials are typically high in complex carbohydrates [10,91,92]. A low-carbohydrate vegan (“Eco-Atkins”) diet has also been shown to decrease body weight and cardio-metabolic risk factors [92]. However, a recent systematic review and meta-analysis of low-carbohydrate diets has not shown any superiority of these diets in the long term in terms of glycemic control, weight, or blood lipids [94]. Therefore, macronutrient distribution should be based on individualized assessment of current eating patterns, preferences, and metabolic goals [93]. Reducing the intake of saturated fat and added sugars while increasing the intake of fiber and complex carbohydrates seems to be a reasonable approach [95].

Fiber contributes to bulk in the diet without adding digestible calories, thus leading to satiety and weight loss. Additionally, soluble fiber binds with bile acids in the small intestines, increasing fecal bile salt excretion and thus reducing cholesterol [96], and reduces blood lipids and blood glucose. High fiber consumption has been linked to reduced body weight, lower blood pressure and blood lipids, reduced plaque formation and cardiovascular risk, and lower risk of type 2 diabetes [97,98,99]. Plant-based diets are also lower in saturated fat and dietary cholesterol. Replacing saturated fat with polyunsaturated and monounsaturated fat has been shown to decrease insulin sensitivity and reduce cardio-metabolic risk, independent of changes in body weight [100,101,102,103,104,105,106].

Vegetable proteins reduce the concentrations of blood lipids [107,108,109,110], reduce the risk of obesity and cardiovascular disease, and may have anti-inflammatory and anti-cancer effects [111,112]. High intake of antioxidants and micronutrients from whole plant foods represents another potential cardio-metabolic beneficial mechanism [113]. Plant sterols that have a structure similar to that of cholesterol reduce the cardiovascular risk and mortality [114,115], have anti-inflammatory effects, and positively affect coagulation, platelet function, and endothelial function [116], as well as glycemic control in patients with type 2 diabetes [117].

It appears that plant-based diets exert cardio-metabolic benefits via several independent mechanisms. When eating all whole plant foods, the synergistic effect may be greater than a mere additional effect of eating isolated nutrients.

## 9. Ensuring Complete Nutrition

Certain nutrients may be less abundant in plant-based diets, compared with diets including animal products, although there is considerable variation depending on specific diet choices. These nutrients include protein, fat (particularly saturated fat), zinc, vitamin D, and vitamin B_12_. However, manifestations of deficiencies are not more common in vegetarian populations than in omnivorous populations [97].

Vitamin B_12_ deserves special attention. It is made by neither plants nor animals but is found in animal products due to its formation by intestinal bacteria. For those following plant-based diets, B_12_-fortified products or supplements ensure that needs for this essential nutrient are met. Other populations who should take a B_12_ supplement, regardless of their dietary pattern, include those who are 50 years or older, those who have digestive disorders such as Crohn’s disease that limit B_12_ absorption, and those who take certain medications such as acid-blockers and metformin [118].

Plant-based diets merit inclusion in dietary recommendations. With guidance and support, such therapeutic diet changes are well-accepted and sustainable, as determined by data on long-term adherence and food acceptability questionnaires [119,120].

The Academy of Nutrition and Dietetics states that, “…appropriately planned vegetarian, including vegan, diets are healthful, nutritionally adequate, and may provide health benefits for the prevention and treatment of certain diseases.” [97]. “Appropriately planned” means that it is important to keep the main sources of macro- and micro-nutrients in mind, and make sure the diet supplies one’s need for all of them.

## 10. Conclusions

Vegetarian diets represent an effective means for the prevention and treatment of cardio-metabolic diseases.

Properly planned vegetarian diets are healthful and effective for weight and glycemic control, and provide metabolic and cardiovascular benefits, including reversing atherosclerosis and decreasing blood lipids and blood pressure. The cardio-metabolic benefits seem to be greater with vegan than lacto-ovo-vegetarian diets [121]. The use of plant-based diets as a means of prevention and treatment of cardio-metabolic disease deserves to be promoted through dietary guidelines and recommendations.
